# Raising Money for Hospitals

**Published:** 1923-02

**Authors:** Frank Inch

**Affiliations:** Secretary. Norfolk and Norwich Hospital, Norwich


Sir,—On page 88 of your issue of January you have inserted a small paragraph stating that " unemployment is so serious in East Anglia that a contributory scheme has been postponed, but it is hoped to prepare it in time for it to be started in 1924, both in town and country."

In order that this paragraph should not give a wrong impression to a large number of hospitals to whom I have given information regarding our contributory scheme which has been in full working order now for over two years, I shall be glad if you will allow me to say that the contributory scheme in connection with this and other hospitals in Norwich, which is organised by the Norwich Hospitals and Medical Institutions Sunday and Saturday Fund, is making most satisfactory progress both in this city and in the County of Norfolk and has contributed very largely to the gratifying financial position of the Norwich hospitals. For the year 1922 this hospital received from this fund £17,880 (an increase of £6,600 over the previous year), the Jenny Lind Children's Hospital £2,570, and the Norfolk and Norwich Eye Infirmary £1,229, in addition to grants made to nursing and other associations.

As you are no doubt aware, the basis of this contributory scheme is that members contribute a minimum sum of 1d. per week, for which free treatment at the hospitals is given in return.

Frank Inch,
Secretary.

Norfolk and Norwich Hospital,
Norwich.

